# Data on investigating the quantitative and qualitative status of effluent in a petrochemical complex in Iran

**DOI:** 10.1016/j.dib.2018.08.174

**Published:** 2018-08-31

**Authors:** Mohammad Hadi Dehghani, Mahnaz Bahram Abadi, Mahmood Alimohammadi, Zoha Heidarinejad

**Affiliations:** a Department of Environmental Health Engineering, School of Public Health, Tehran University of Medical Sciences, Tehran, Iran; b Institute for Environmental research, Center for Solid Waste Research, Tehran University of Medical Sciences, Tehran, Iran; c Islamic Azad University, Science and Research Branch, Faculty of Environment & Energy, Department of Environmental Engineering, Tehran, Iran; d Food Health Research Center, Hormozgan University of Medical Sciences, Bandar Abbas, Iran

**Keywords:** Wastewater, Environmental standards, Petrochemical complex, Industrial effluent

## Abstract

The aim of this data was investigating the quantity and quality of the produced effluent by different petrochemical industry units in Iran and comparison of effluent with the present standards. In the present data, 5 effluent channel of the complex with interval of 12 h (in two shifts) were sampled and 28 physical and chemical parameters were analyzed according to the standard methods. These parameters are pH, Temperature, DO, Conductivity, Color, TDS, TSS, TP, PO_4_^3^^−^, Oil, BOD_5_, COD, Turbidity, TKN, Fe, Ca^2+^, Mg^2+^, Cl^−^, SO_4_^2^^−^, Si^4+^, CO_3_^2−^, HCO_3,_ NO_2_^−^, NO_3_^−^, NH_3_, Na, K^+^, Mn^2+^. Then, the average of each parameter was obtained for each channel, and finally, values of these parameters were compared with the standard set by Iranian Environmental Protection Agency for discharge to surface water resources. Gathered Data showed that many of these parameters, including Oil, BOD_5_, COD, Turbidity, PO_4_^3−^, SO_4_^2−^, TSS, in effluent of industrials are higher than the permitted amount. Therefore, regarding discharge of the to the surface water (seawater) and in accordance with Environment Protection Agency standards for effluent disposal, it should be purified to about 90% prior to discharge. Due to high concentration of solutes in petrochemical wastewater, it is not possible to use it for agricultural purpose. In this data, due to ethical considerations, we did not mention the name of petrochemical complex.

**Specifications table**

**Table:** 

Subject area	Environmental Science
More specific subject area	Industrial Effluent
Type of data	Table and Figure
How data was acquired	Five effluent channel of the complex with interval of 12 h (in two shifts) were sampled from the wastewater and 28 physical and chemical parameters were analyzed. The parameters include pH with pH meter device (HACH (HQ 40d)), temperature (by Thermometer), EC with EC meter device (HACH (HQ 40d)), TDS with TDS meter device (HACH (HQ 40d)), Fe and Mn were measured with flame atomic absorption spectrophotometer (Younglin AAS 8020).
Data format	Raw, Analyzed
Experimental factors	The physical and chemical parameters of wastewater were analyzed according to standard method for water and wastewater experiments.
Experimental features	An average of parameters was obtained in effluent from each channel, values of these parameters were compared with the standard set by the Iranian Environmental Protection Agency for discharge to surface water resources.
Data source location	Iran
Data accessibility	The data are available with this article
Related research article	*Not applicable*

**Value of the data**

• In this data, analysis of physical and chemical parameters of industrial effluent in petrochemical industry has been investigated.
• Results of this data can be used to show that many of the physical and chemical parameters of petrochemical effluent exceed standards.
• The data are useful in demonstrating that where there are high amounts of solute in effluent of petrochemical industry, reuse of treated wastewater is not possible for irrigation of green and agricultural space.

## 1 Data

**Fig. 1:**
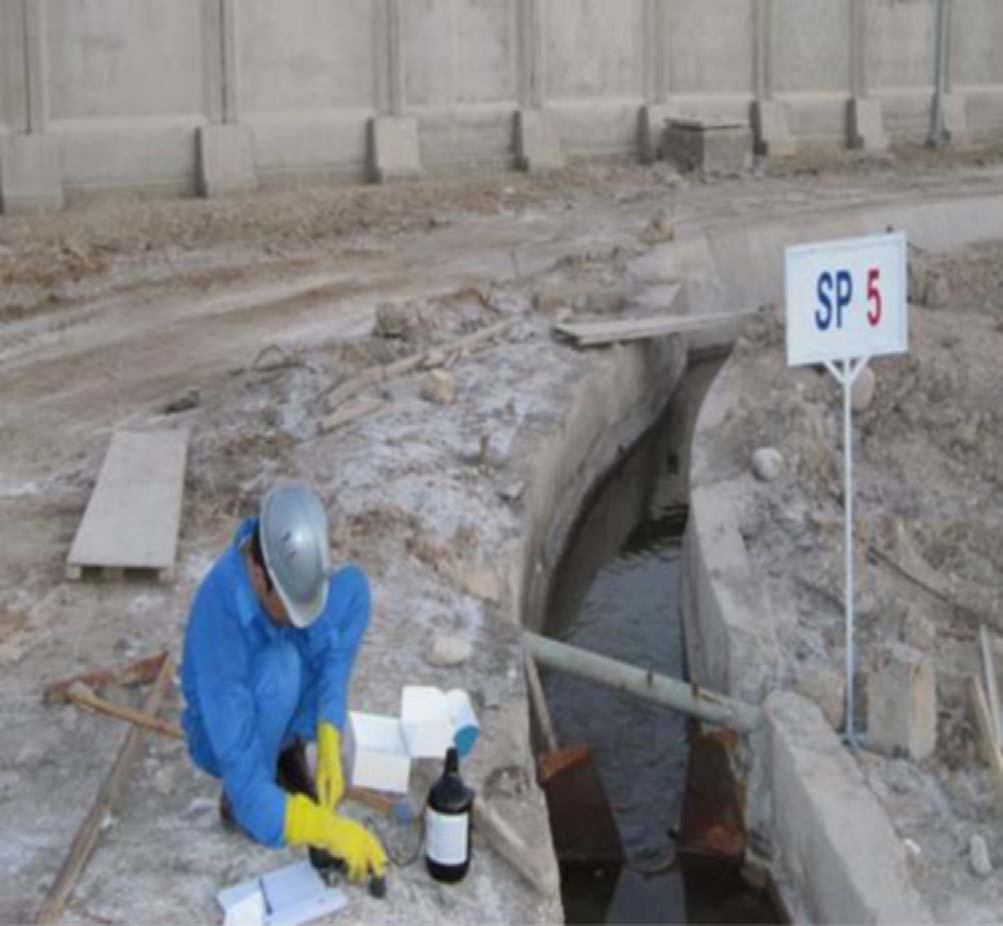
Images of sampling site from some of petrochemical complex channels.

**Fig. 2:**
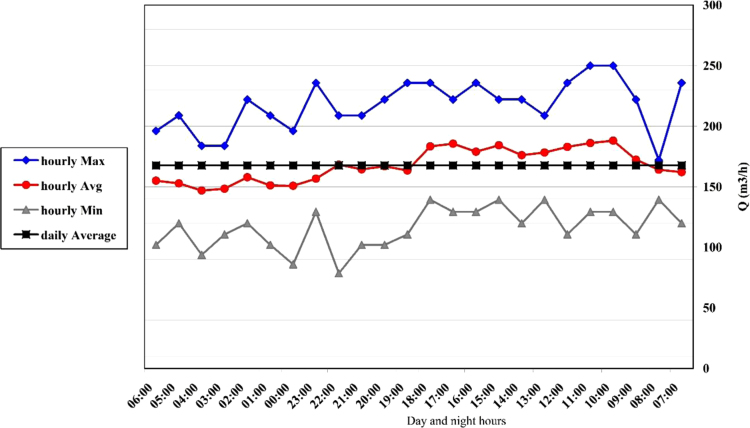
Changes in hourly flow rate at channel 1.

**Fig. 3:**
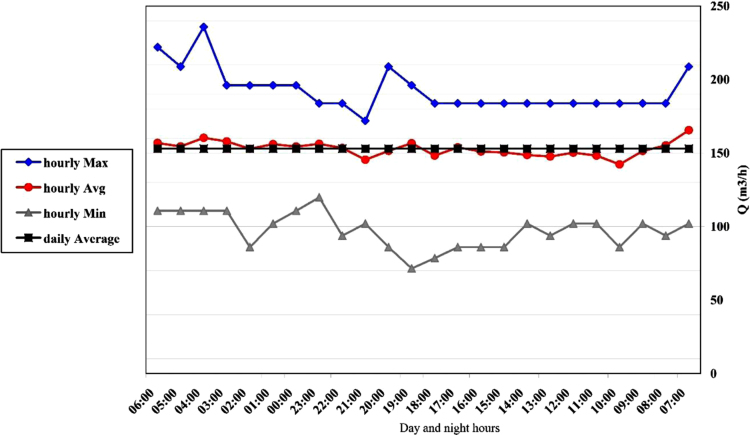
Changes in hourly flow rate at channel 2.

**Fig. 4:**
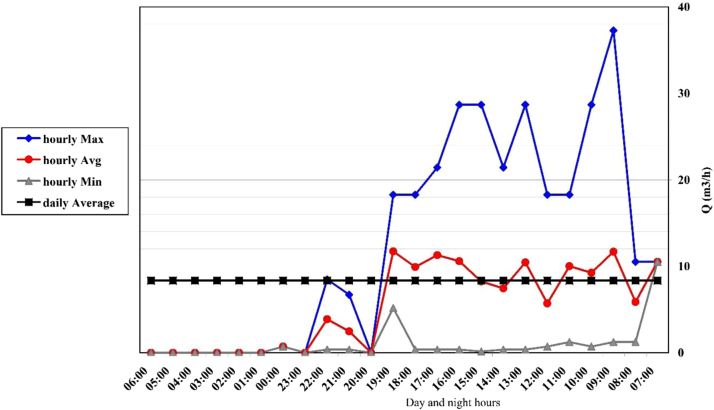
Changes in hourly flow rate at channel 3.

**Fig. 5:**
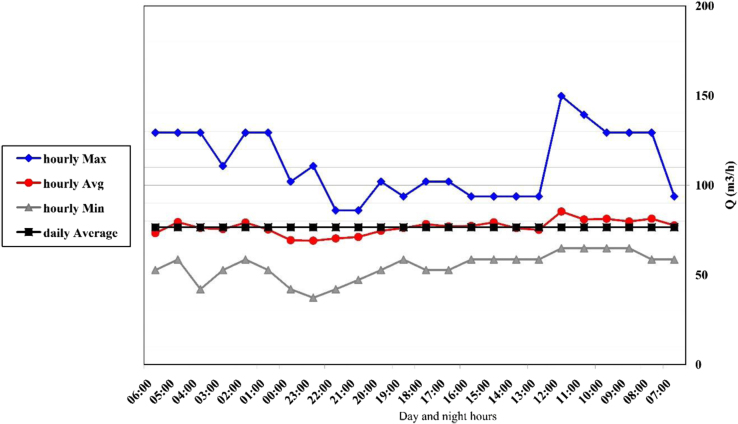
Changes in hourly flow rate at channel 4.

**Fig. 6:**
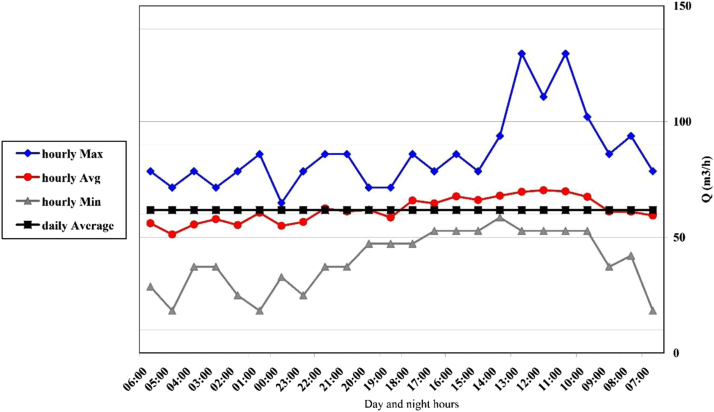
Changes in hourly flow rate at channel 5.

Fig. 1 shows the images of sampling site from some of petrochemical complex channels. Specification of the existing channels in petrochemical complex area in Table 1. Code of testing method of qualitative parameters of wastewater according to standard method book shows in the Table 2. Changes in hourly flow rate at channels 1–5, shows in the Fig. 2, Fig. 3, Fig. 4, Fig. 5, Fig. 6. Table 3 shows, comparison of the final channel with effluent discharge standards from environmental protection agency. Comparison of contaminant parameters with surface water discharge standards shows in the Table 4. Table 5 shows comparison of parameter (pollutants) with discharge standard to be absorbent well and Table 6 shows, comparison of pollutant parameters with standards of use in agriculture and irrigation.

**Table 1:** Specification of the existing channels in petrochemical complex area.

Title channel number	1	2	3	4	5
Dimensions (Width, height)	163 × 121	1239 × 119	159 × 113	131 × 193	95 × 90
Transitional flow rate (m^3^/h)	250	236	37	150	129

**Table 2:** Code of testing method of qualitative parameters of wastewater according to standard method book (1).

**Parameter**	**Test procedure code**
pH	pH Meter (HACH (HQ 40d))
Temperature	Thermometer
DO	HQ40d
EC	EC Meter ( HACH (HQ 40d))
Color	VISUAL
TDS	TDS Meter ( HACH (HQ 40d))
TSS	Standard Method 22th Edition, 2540
TP	ISO 6878
PO_4_^3−^	ISO 6878
Oil	ASTM D 4281-95
BOD_5_	Standard Method 22th Edition, 5210B(OXITOP WTW)
COD	ISO 15705
Turbidity	ASTM D 188900
TKN	Standard method 4-500-
Fe	AAS
Ca^2+^	ASTM D 511-03
Mg^2+^	ASTM D 511-03
Cl^−^	ASTM D 512-89 B
SO_4_^2−^	ASTM D 516-88
Si^4+^	Standard method 4500-SIO_2_
CO_3_^2−^	Standard Method 22th Edition, 2320B
HCO_3_^−^	Standard Method 22th Edition, 2320B
NO_2_^−^	Standard method 4500 B
NO_3_^−^	SPECTRO PHOTOMETER HACH KIT
NH_3_	ASTM D 142603
Na^+^	AES
K^+^	AES
Mn^2+^	AAS

**Table 3:** Comparison of the final channel with effluent discharge standards from environmental protection agency [1], [2], [3], [4], [5], [6], [7].

Parameter	Unit	Channel 1	Channel 2	Channel 3	Channel 4	Channel 5	Output channel	Discharge to surface water	Discharge into absorbent well	Agricultural and irrigation use
pH	–	9.16	9.23	7.03	9.18	8.08	8.53	6.5–8.5	5–9	6–8.5
Temperature	°C	21.5	21.16	18.61	19.5	19.03	19.95	(Note 4)	–	–
DO	mg/l	8.05	7.45	8.35	8.2	7.6	7.93	2	–	2
EC	μs/cm	5725	5590	5427	4013	4863	5123	–	–	–
Color		–	–	–	–	–	–	–	–	–
TDS	mg/l	3400	3064	2987	2299	2567	2863	(Note 1)	(Note 2)	–
TSS	mg/l	475	621	1018	1924	437.5	895	40	40 (Moment 60)	100
TP	mg/l	9.09	13.6	2479	4.76	12.9	504	–	–	–
PO_4_^3−^	mg/l	8.17	12.96	4299	4.4	11.6	867	18.4	18.4	–
Oil	mg/l	964	248	138	250	53	303	10	10	10
BOD_5_	mg/l	52.3	57.84	58	70.6	51	57.9	30(Moment 60)	30(Moment 60)	10
COD	mg/l	280.6	226	133	231	232	220	60(Moment 100)	60(Moment 100)	200
Turbidity		54	86.66	112	541	93	177	50	–	50
TKN	mg/l	210	30.7	10.68	17	28	59	–	–	–
Fe	mg/l	1.25	3.69	7.55	21.7	4.4	7.7	3	3	3
Ca^2+^	mg/l	344	262	799	232	340.8	395	75	–	–
Mg^2+^	mg/l	328	174	414	282	248	289	100	100	100
Cl^−^	mg/l	2537	1573	1707	1362	1558	1747	600 (**Note 1**)	600(**Note 2**)	600
SO_4_^2−^	mg/l	848	849	1356	1068	1101	1044	400(**Note 1**)	400(**Note 1**)	500
Si^4+^	mg/l	5.95	5.7	12.58	8.72	6.09	7.8	–	–	–
CO_3_^2−^	mg/l	292	745	190	344	17.3	317.7	–	–	–
HCO_3_^−^	mg/l	49.7	218	62.57	534	41.5	181	–	–	–
NO_2_^−^	mg/l	0.153	0.32	0.19	5.02	0.22	1.18	10	10	–
NO_3_^−^	mg/l	21.2	24.2	27.2	35.5	16.5	24.9	50	10	–
NH_3_	mg/l	222	996	221	55.68	9.13	300.7	–	–	–
Na^+^	mg/l	495	300	337	239	305	335	–	–	–
K^+^	mg/l	9.95	5.2	2.34	5.06	7	5.9	–	–	–
Mn^2+^	mg/l	0.05	0.06	0.06	0.085	0.05	0.06	1	1	1

Note 1: Discharged with concentration above the specified level in the table is allowed if the effluent, do not increase chloride, sulfate concentration more than 10% in 200-m radius.

Note 2: Discharge above specified concentration in the table, is permitted if augmentation of chloride, sulfate and TDS wastewater do not exceed more than 10% of water consumption.

Note 3: Existing industries will be allowed to reduce BOD_5_ and COD by at least 90%.

Note 4: The temperature should not reduce or increase temperature of receiving source more than 3 °C at radius of 200 m from its entrance.

**Table 4:** Comparison of contaminant parameters with surface water discharge standards.

**Parameter (Pollutants)**	**Compare with the discharge absorbent well standard**
TSS	22.3 times more than standards
Oil	30 times more than standards
BOD_5_	1.9 times more than standards
COD	3.6 times more than standards
Fe	2.6 times more than standards
Mg^2+^	2.9 times more than standards
Cl^−^	2.9 times more than standards
SO_4_^2−^	2.6 times more than standards

**Table 5:** comparison of parameter (pollutants) with discharge standard to be absorbent well.

**Parameter (Pollutants)**	**Comparison with dispose of surface water standards**
TSS	22.3 times more than standards
Oil	30 times more than standards
BOD_5_	1.9 times more than standards
COD	3.6 times more than standards
Turbidity	1.5 times more than standards
Fe	2.6 times more than standards
Ca^2+^	5.3 times more than standards
Mg^2+^	2.9 times more than standards
Cl^−^	2.9 times more than standards
SO_4_^2−^	2.6 times more than standards

**Table 6:** Comparison of pollutant parameters with standards of use in agriculture and irrigation.

**Parameter (Pollutants)**	**Comparison with standards of use in agriculture and irrigation**
TSS	8.9 times more than standards
Oil	30 times more than standards
BOD_5_	1.1 times more than standards
COD	1.5 times more than standards
Fe	2.6 times more than standards
Mg^2+^	2.9 times more than standards
Cl^−^	2.9 times more than standards
SO_4_^2−^	2.2 times more than standards

## 2 Experimental Design, Materials and Methods

### 2.1 Current status of effluent disposal in petrochemical industry

At the time of carrying out this data, five separate concrete channels were used to collect and dispose effluent of different units of petrochemical industry. Now, the industrial effluent flowing in to each of these channels are separately discharged at the end of channels. Mentioned channels in this complex are numbered from 1 to 5, each of them has different dimension and size and the amount of flow by each of these channel is different. Information about these channels is given in Table 1. It should be noted that these channels have a rectangular section and are concrete type.

At the end of the channel routes number 1 to 5 in terms of positioning in petrochemical industry are as follows: 1, 2, 3 channels are on the south side and 4, 5 channels are located on the western and northern sides of industry respectively.

### 2.2 Sampling site

The aim of this data was to provide investigated quantitative and qualitative characteristics of 4 industrial effluent channels in order to concentrate them at a point for final purification. In Fig. 1, images of sampling sites from some of petrochemical complex channels are observed.

### 2.3 Method

Quantitative and qualitative measurements of effluent were carried out over a period of 15 consecutive days in two 12 h shifts (Shift 1: from 7 a.m. to 7 p.m., shift 2: 7 a.m. to 7 a.m.).

Samples were transferred to the laboratory at the end of each shift and measured results were recorded. Measurement of effluent discharge, DO, Temperature and pH was performed at the sampling site. At the start of each shift (before sampling) sampling containers should be completely washed with Deionized water.

Sampling and evaluation of required parameters in wastewater outlet, was carried out hourly. Sampling of each channel performed completely separate and each tie 200 cc of sample was taken from sampling site and was poured in 2. Littre glass container (storage container), (12 samples of 200 cc in each shift and total 2400 cc from each station). Parameters that were checked at any point included CO_3_^2−^, HCO_3_^−^_,_ TDS, Cl, SO_4_^2−^_,_ Ca^2+^, Mg^2+^, Fe, Mn, Na^+^, K^+^, Si^4+^, Turbidity, Color, Temperature, pH, EC, TSS, Oil, Grease, DO, BOD_5_, COD, PO_4_^3^^−^, TP, NO_3_^−^_,_ NO_2_^−^, NH_3_,TKN.

Each time of sampling from stations, temperature, DO and pH of each sample were measured before sample was discharged into the containers. Sample containers were encoded and the corresponding code was recorded in the forms. Samples transferred to the laboratory were analyzed at the time and on the same day and their holding time were considered. All experiments were performed according to standard method. In Table 2 code of wastewater experiment methods are provided:

### 2.4 Hydraulic foundation

In this data, in order to obtain quantitative bases, flow measurement was carried out in addition to qualitative sampling. After installing overflows in desired location and before taking samples from the site (for transferring to the laboratory for quantitative analysis), the exact height of water on the overflow was measured and inserted by a metal ruler.

#### 2.4.1 Channel 1

The range of discharge variation in this channel was 79–250 m^3^/h with an average of 165 m^3^ per hour. In Fig. 2, you can see the changes per hour.

#### 2.4.2 Channel 2

The range of discharge variation in this channel was 71–236 m^3^/h with an average of 153 m^3^ per hour. In Fig. 3, you can see the hourly change.

#### 2.4.3 Channel 3

The range of discharge variation in this channel was 0–37 m^3^/h with an average of 8 m^3^ per hour. In Fig. 4, you can see the hourly change.

#### 2.4.4 Channel 4

The range of discharge variation in this channel was 18–129 m^3^/h with an average of 62 m^3^ per hour. In Fig. 5, you can see the hourly change.

#### 2.4.5 Channel 5

The range of discharge variation in this channel was 18–129 m^3^ per hour with an average of 62 m^3^/h. In Fig. 6, you can see the hourly change.

At present, industrial effluent from channels 1 to 5 of this complex is discharged into the surface water. Many of these pollutant, including oil, COD, BOD_5_, Turbidity, SO_4_^2−^, PO_4_^3−^, TSS, etc are more than wastewater drainage. Therefore, due to the use of effluent of this Petrochemical Complex and its discharge to surface water (seawater) and according to with the standards of the environment protection agency for effluent disposal, effluent should be purified to about 90% to be dischargeable. Due to the high concentration of solutes in this industrial complex, it is not possible to use it for agricultural purposes. In Table 3, the output of the petrochemical complex is compared with effluent discharge standards provided by the environmental protection agency (in accordance with article No. 5 of the water pollution prevention regulation of 30/11/1994).

Total number of samples taken during 14 days was 150 cases that on each sample 28 pollutant parameters analyzed and compared with the results obtained from outlet channel with wastewater discharge standards from the environmental protection agency.
